# C-terminal intrinsically disordered region-dependent organization of the mycobacterial genome by a histone-like protein

**DOI:** 10.1038/s41598-018-26463-9

**Published:** 2018-05-29

**Authors:** Anna Savitskaya, Akihito Nishiyama, Takehiro Yamaguchi, Yoshitaka Tateishi, Yuriko Ozeki, Masaaki Nameta, Tomohiro Kon, Shaban A. Kaboso, Naoya Ohara, Olga V. Peryanova, Sohkichi Matsumoto

**Affiliations:** 1 Department of Bacteriology, Niigata University School of Medicine, Niigata, Japan; 2 Electron Microscope Core Facility, Niigata University, Niigata, Japan; 3 Russia-Japan Center of Microbiology, Epidemiology and Infectious Diseases, Krasnoyarsk State Medical University, Krasnoyarsk, Russia; 4 Department of Pharmacology, Osaka City University Medical School, Osaka, Japan; 5 Department of Oral Microbiology, Okayama University Graduate School of Medicine, Dentistry and Pharmaceutical Sciences, Okayama, Japan

## Abstract

The architecture of the genome influences the functions of DNA from bacteria to eukaryotes. Intrinsically disordered regions (IDR) of eukaryotic histones have pivotal roles in various processes of gene expression. IDR is rare in bacteria, but interestingly, mycobacteria produce a unique histone-like protein, MDP1 that contains a long C-terminal IDR. Here we analyzed the role of IDR in MDP1 function. By employing *Mycobacterium smegmatis* that inducibly expresses MDP1 or its IDR-deficient mutant, we observed that MDP1 induces IDR-dependent DNA compaction. MDP1-IDR is also responsible for the induction of growth arrest and tolerance to isoniazid, a front line tuberculosis drug that kills growing but not growth-retardated mycobacteria. We demonstrated that MDP1-deficiency and conditional knock out of MDP1 cause spreading of the *M*. *smegmatis* genome in the stationary phase. This study thus demonstrates for the first time a C-terminal region-dependent organization of the genome architecture by MDP1, implying the significance of IDR in the function of bacterial histone-like protein.

## Introduction

Histone is a primary component of the eukaryotic nucleosome, where DNA wraps around an octamer of four core histones (two of each histone H2A, H2B, H3, and H4). Linker histones (H1 and H5) bind to the nucleosome and adjacent linker DNA. These histones possess intrinsically disordered regions (IDR), which lack typical secondary and tertiary structures^1^. Discoveries of IDR and their high frequency in eukaryotic proteins, especially nuclear proteins^2,3^, have been challenging the doctrine of traditional structure-function paradigm. For instance, polycationic C-terminal IDR of histone H1 binds to DNA or negatively charged protein domains. The interaction with the targets induces the secondary structures (e.g., α-helixes) in IDR, resulting in the formation of stable folding and oligomerization of chromatin fibers^1,4–7^. IDR of the histones play significant roles in genome functions through chromatin condensation, leading to regulation of transcription, replication, and recombination^8–10^.

The bacterial chromosome is also folded into a compact structure, called nucleoid. Bacterial histone-like proteins, such as HU (initially reported as a heat stable DNA-binding protein of *Escherichia coli* strain U93)^11^, integration host factor (IHF)^12^, and histone-like nucleoid structuring protein (H-NS)^13^ are small basic proteins (10–15 kDa), which share functions with histones, especially in the DNA-compaction capacity^14,15^. HU is one of the most extensively studied histone-like proteins and has been found to be conserved in all sequenced eubacteria^11,14–16^. It exists as a hetero- or homodimer (e.g., HUαα and HUαβ in *E*. *coli*) and organizes DNA conformation by bending DNA and producing a superhelical turn. However, IDR is rare in bacterial proteins^2,3^. As such, unlike eukaryotic histones, bacterial histone-like proteins generally lack IDR.

**Figure 1:**
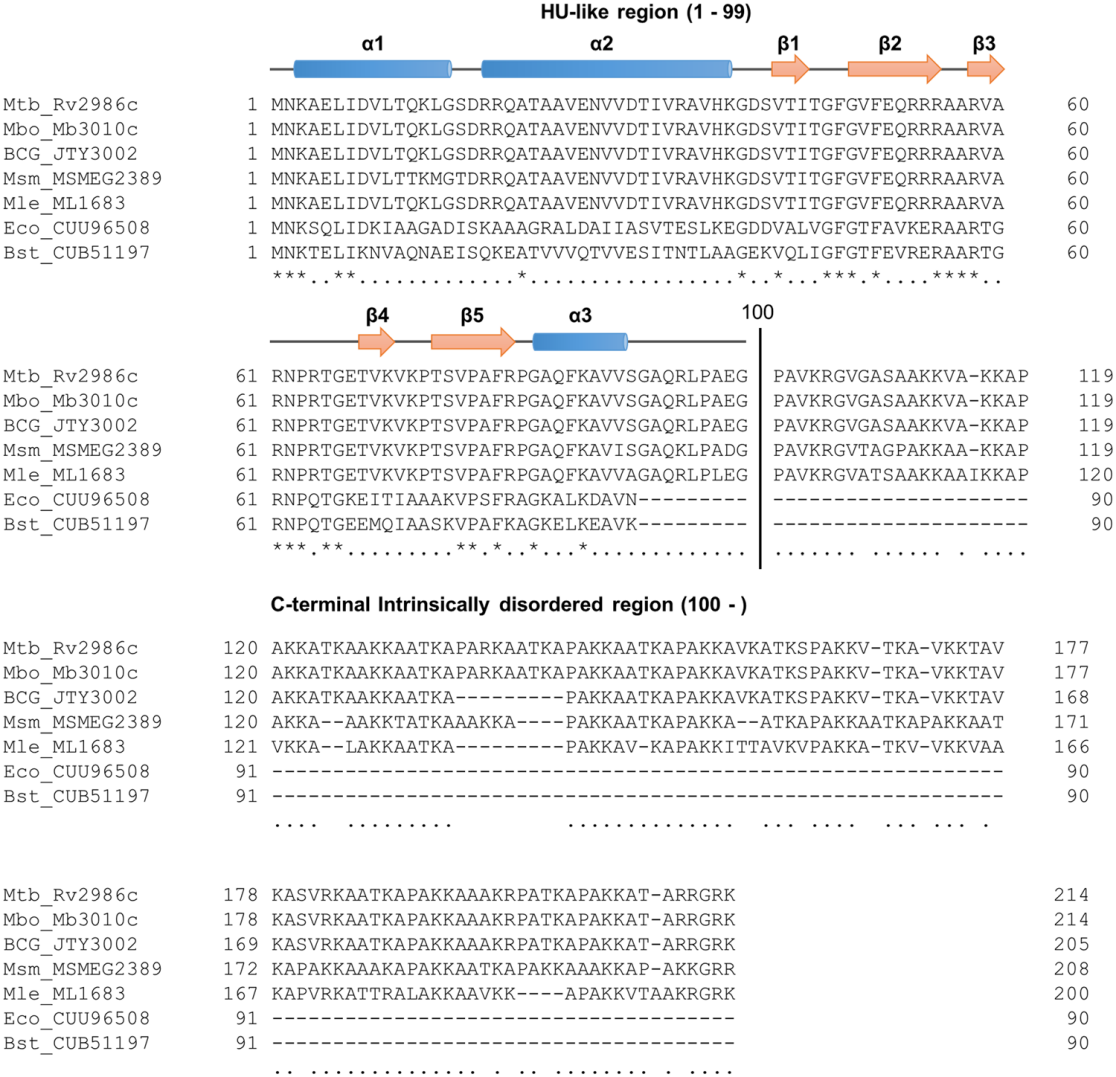
Alignment of amino acid sequences of MDP1 from mycobacterial species and HU. Amino acid sequences of MDP1 in 4 mycobacterial species, a BCG vaccine strain, *E*. *coli* HU and *Bacillus subtilis* HU are aligned. Secondary structures of the N-terminal 99 amino acid region of MDP1_Mtb_^19^ are presented as α helices (indicated as tubes) and β strands (indicated as arrows) at the top of the alignment. The black vertical line between 99th and 100th amino acid residues indicates a boundary of NTD and C-terminal IDR which was suggested previously^19^. Aligned sequences are as follows: Mtb_Rv2986c, *M*. *tuberculosis* H37Rv MDP1 (Rv2986c, MDP1_Mtb_); Mbo_Mb3010c, *M*. *bovis* AF2122/97 MDP1 (Mb3010c); BCG_JTY3002, BCG strain Tokyo MDP1 (JTY3002); Msm_MSMEG_2389, *M*. *smegmatis* mc^2^_155 MDP1 (MSMEG2389); Mle_ML1683, *Mycobacterium leprae* TN MDP1 (ML1683); Eco_CUU96508, *E*. *coli* 10270 HUβ (CUU96508); and Bsu_CUB51197, *B*. *subtilis* JRS10 HU (CUB51197).

Mycobacteria are nonmotile, nonsporulating, acid-fast bacilli. There are a lot more than 100 known species. Although most of mycobacteria are commonly found in wild animals or in the environment, some are human pathogens for instance, *Mycobacterium tuberculosis* and *Mycobacterium leprae*. Mycobacteria are slow growing bacteria and some human pathogens can reside in a host for long period without sterilization by the immune responses. All the examined mycobacterial species produce a unique histone-like protein designated Mycobacterial DNA-binding protein 1 (MDP1), which contains a long IDR unlike histone-like proteins in other bacteria. The molecular weights of MDP1 are therefore larger than common histone-like proteins (e.g., 22 kDa in *M*. *tuberculosis* and 21 kDa in *Mycobacterium smegmatis*) (Fig. 1)^14,17,18^. The N-terminal 99-amino acid domain (NTD) of MDP1 is HU-like and highly conserved in mycobacterial species^14,17–19^. X-ray crystallography of NTD of *M*. *tuberculosis* MDP1 (MDP1_Mtb_) has revealed a repeat of two α-helices at the N-terminus (contributing to dimerization), followed by a β-sheet domain which forms a DNA-binding arm (Fig. 1)^19^. In contrast, more than 100-amino acid C-terminal domain possesses characteristics of IDR that is rich in Lys, Ala, and Pro residues and composed of short sequence repeats such as, PAKK^1,14,17^. Except for a few α-helices, there is no typical secondary structure in the C-terminal half from Pro^100^ of both MDP1_Mtb_ and *M*. *smegmatis* MDP1, as shown by PSIPRED, the structure prediction software (Supplementary Fig. S1).

MDP1 is an abundant protein in mycobacterial cells and is considered to play pivotal roles in mycobacterial genome functions. In the previous study, we found suppressive effects of MDP1 on the macromolecular biosynthesis of DNA and RNA in cell-free assay and bacterial growth under MDP1 overexpression^18,20^. Mukherjee *et al*. later showed that overexpression of MDP1 induced nucleoid condensation^21^. In addition, the down-regulation of replication and cellular metabolism by MDP1 was also suggested to contribute to the long-term survival of mycobacteria^22^.

As for the IDR function of MDP1, it was reported that the intact structure of MDP1 is required for high-affinity binding to short strand DNA and inhibitory effects on the RecA-mediated modulation of DNA topology in cell-free assay^21,23^. On the other hands NTD or C-terminal IDR alone showed only limited affinity to DNA. These *in vitro* studies implicated the importance of IDR in the function of MDP1. In order to know more precise functions of MDP1-IDR, we created genomic MDP1-gene-deficient *M*. *smegmatis* strains, which inducibly express intact MDP1 or only NTD of MDP1, and MDP1-conditional knock-down *M*. *smegmatis* strains. By employing these strains, we observed that IDR of MDP1 is critical for MDP1-related phenotypes of *M*. *smegmatis* cell, including genome compaction, suppression of DNA synthesis, and drug tolerance to isoniazid, a front line tuberculosis drug. This study provides a reasonable basis for elucidation of IDR function in bacteria and its evolution in living organisms.

## Results

### Construction of *M*. *smegmatis* mc^2^_155 strains constitutively or inducibly expressing intact MDP1 or NTD of MDP1 (NTD)

MDP1 gene (designated as *mdp1*) is dispensable in *M*. *smegmatis*, unlike in *M*. *tuberculosis* where it is reported to be essential^24,25^. MDP1-deficient *M*. *smegmatis* (Δ*mdp1*) and its complemented strain have been used to investigate *in vivo* MDP1 functions^22,26,27^. In order to know the function of the C-terminal IDR of MDP1, we first constructed plasmids to complement Δ*mdp1* by whole MDP1 or its C-terminal IDR deletion mutant (NTD). However, we found remarkable growth delay of the bacteria after transformation with the plasmid to express whole MDP1 gene by its own promoter or constitutively active *P*_*smyc*_ promoter^28^ (Supplementary Fig. S2A, *mdp1*). Under such a condition, we observed no or a small number of bacterial colonies on the agar but MDP1 expression by obtained colonies was low as judged by SDS-PAGE and western blot analysis (Supplementary Fig. S2B). By contrast, we observed a normal growth rate of Δ*mdp1* transformed with a plasmid that expresses NTD (Supplementary Fig. S2A, *NTD*) by its own promoter or *P*_*smyc*_ promoter. This agreed with the growth suppressive effects of whole MDP1, as we previously indicated^20^, implying the importance of IDR on this growth suppressive effect. However, this data suggested the limitation of constitutive expression of MDP1 for analysis of its function.

Because of the limitation of analysis of MDP1-constitutively expressing bacteria, next we constructed *M*. *smegmatis* inducibly expressing intact MDP1 or NTD. For this purpose, we used acetamidase gene promoter/regulator system cloned from *amiCADSE* locus of *M*. *smegmatis* (designated as AMI)^29^. DNA sequences of Histidine (His)-tagged intact MDP1 (*mdp1*) or NTD (*NTD*) were cloned downstream of AMI and introduced into Δ*mdp1*^26^. Finally, the strains inducibly expressing intact MDP1 (AMI-*mdp1*) or NTD (AMI-*NTD*) were selected.

We first examined the optimal concentration of acetamide (Ace) to induce the expression of MDP1 and NTD. After 24 h incubation of AMI-*mdp1* and AMI-*NTD* with the various concentrations of Ace, MDP1 expression was detected by western blot analysis. As shown in Supplementary Fig. S3A, both MDP1 and NTD expression were induced in response to Ace addition. Dose-dependency of the expression was not significant up to 0.2% Ace. His-tagged MDP1 and NTD whose deduced molecular weights based on their primary sequences are 22.0 kDa and 11.5 kDa, respectively, were detected as approximately 28 kDa and 13 kDa respectively, in the SDS-PAGE analysis. At 0.2% Ace, both AMI-*mdp1* and AMI-*NTD* presented similar amounts of MDP1 and NTD (approximately 62 ng and 52 ng in 10 µg of total proteins, respectively; Supplementary Fig. S3A). In the following experiments, final 0.2% Ace concentration was used to induce protein expression.

We next examined the optimal induction time of MDP1 and NTD in the presence of 0.2% Ace. As shown in Supplementary Fig. S3B, MDP1 expression increased in a time-dependent manner up to 72 h, whereas NTD expression was almost saturated at 12 h.

### MDP1 causes growth retardation of *M*. *smegmatis* in an IDR-dependent manner

**Figure 2:**
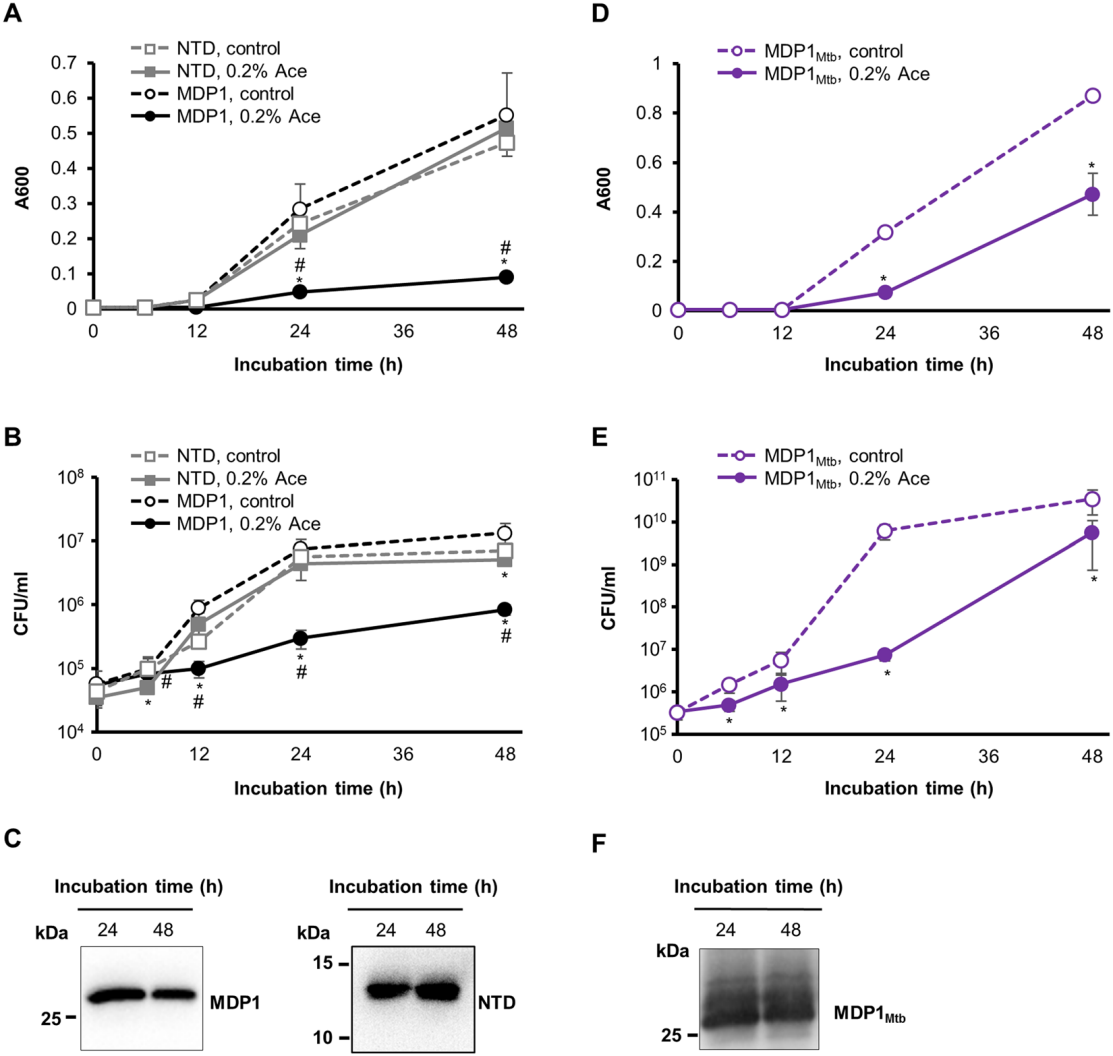
Growth kinetics of MDP1- and NTD- expressing *M*. *smegmatis*. (**A**,**B**) Growth curves of AMI-*mdp1* and AMI-*NTD* in the presence or absence of 0.2% Ace are indicated as the absorbance at 600 nm (A600) (**A**) and colony-forming units (CFUs) per ml (CFU/ml) (**B**) Ace was added to the culture at 0 h. Solid black line with closed circle, dashed black line with open circle, solid gray line with closed square, and dashed gray line with open square represents AMI-*mdp1* (+0.2% Ace), AMI-*mdp1* (−Ace, control), AMI-*NTD* (+0.2% Ace), and AMI-*NTD* (−Ace, control) respectively. Data is presented as mean ± SD (n = 3). * and ^#^ *P* < 0.05 compared to control and NTD (0.2% Ace), respectively, at each time point. The result shown is representative of at least two experiments. (**C**) Western blot analysis of intact MDP1 and NTD expression 24 and 48 h after addition of Ace (**A,B**). Full length western blots of the panels shown here were also presented as Supplementary Figure S4A and B. (**D**,**E**) Growth curves of AMI-*mdp1*_*Mtb*_ in the presence or absence of 0.2% Ace are indicated as the A600 (**D**) and CFU/ml (E) Ace was added to the culture at 0 h. Solid line with closed circle and dashed line with open circle represent AMI-*mdp1*_*Mtb*_ (+0.2% Ace) and AMI-*mdp1*_*Mtb*_ (−Ace, control) respectively. Data is presented as mean ± SD (n = 3). *P < 0.05 compared to control at each time point. The result shown is representative of at least two experiments. (**F**) Western blot analysis of MDP1_Mtb_ expression during the growth curve (**D**,**E**). A full length western blot of this panel was also presented as Supplementary Figure S4C.

We investigated the impact of MDP1 and NTD expression on the growth of *M*. *smegmatis*. After bacterial culture was diluted, 0.2% Ace was added to induce MDP1 expression (0 h, Fig. 2) and further incubated for 48 h. Compared to its control, the growth delay of *M*. *smegmatis* was observed by the induction of the intact MDP1. In contrast, NTD-expressing *M*. *smegmatis* showed a similar growth curve to that of control (Fig. 2A,B). The generation time of MDP1-expressing *M*. *smegmatis* was three times slower than those of its control or NTD-expressing *M*. *smegmatis*. We confirmed the expression of MDP1 proteins by western blot at the time points when the growth delay was observed (Fig. 2C).

We also cloned MDP1 gene (*mdp1*_*Mtb*_; Rv2986c) from *M*. *tuberculosis* and constructed its inducible expressing *M*. *smegmatis* (AMI-*mdp1*_*Mtb*_). Inducible MDP1_Mtb_ expression similarly caused growth retardation of *M*. *smegmatis* (Fig. 2D–F), showing the similar function of MDP1_Mtb_ in growth arrest of mycobacteria.

**Figure 3:**
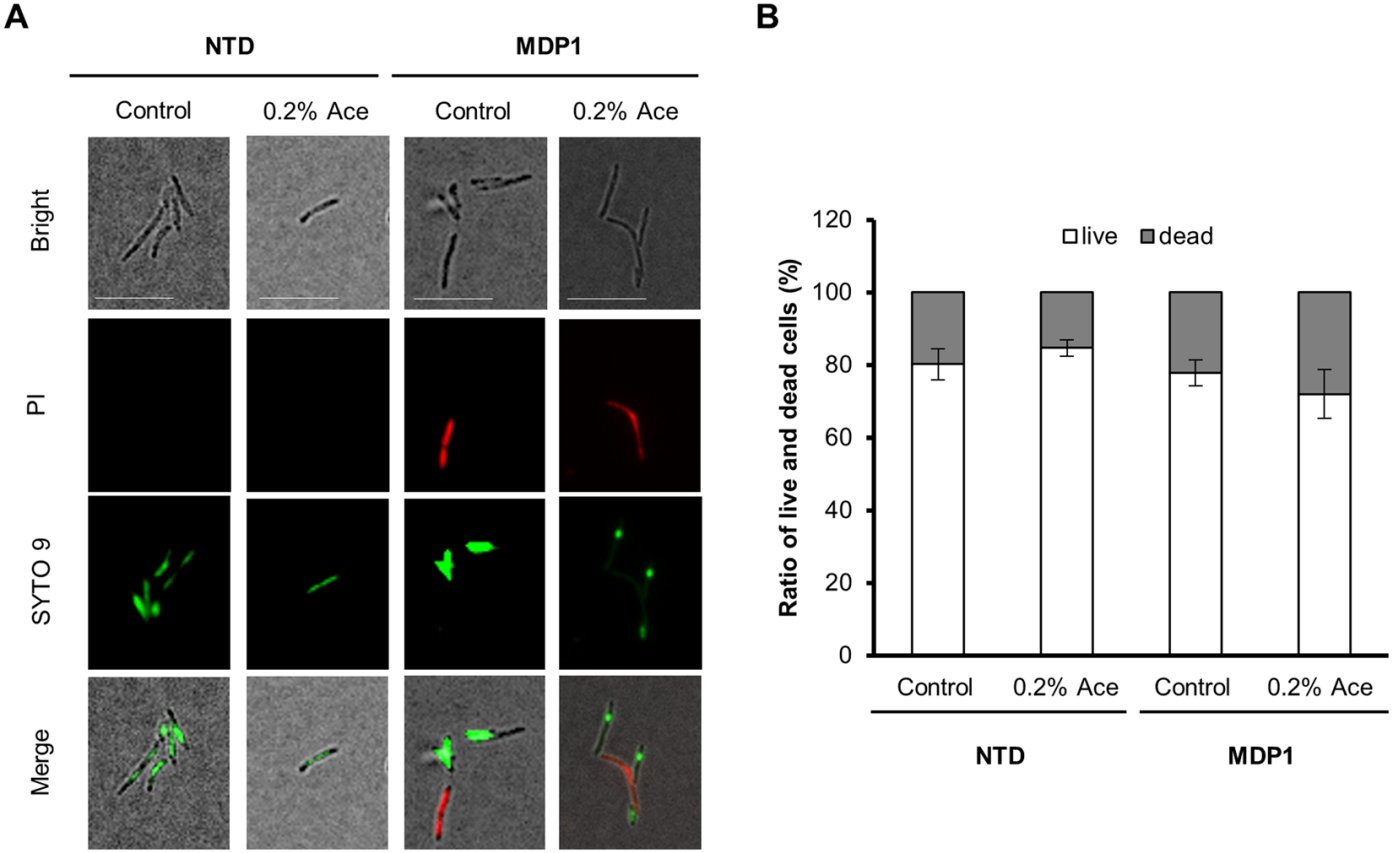
Viability of *M*. *smegmatis* strains in the presence or absence of Ace. (**A**) Fluorescence microscopic images of live (green) and dead (red) bacteria are shown. AMI-*mdp1* (MDP1) and AMI-*NTD* (NTD) cultured in the presence or absence of 0.2% Ace were stained with SYTO 9 (green) and propidium iodide (PI, red) using a Live/Dead BacLight Bacterial viability kit and examined by fluorescence microscopy. Representative cell images in each group are shown. Scale bars, 10 µm. (**B**) Using microscopic images detected in A, percentages of the live and dead cells (white and gray, respectively) were determined in five views (more than 150 cells in each view). Data from a single experiment is presented as mean ± SD of the results of five views. The result shown is representative of at least three experiments.

In *E*. *coli*, H-NS binds to AT-rich regions on the genome and suppresses a wide variety of genes located around its binding regions by bridging adjacent regions^30,31^. It has been reported that overexpression of H-NS causes bacterial death^32^. Based on this knowledge, to access whether growth retardation was caused by the physiological function of MDP1 or bacterial cell death by the artifacts of inducible expressions, we examined the viability of the bacterial cells. Twenty-four hours after adding Ace, bacterial cells were stained with Live/Dead BacLight Bacterial viability kit (Thermo Fisher Scientific, Waltham, MA) (Fig. 3A), and the ratio of live to dead cells in the bacterial population was calculated. The viability of MDP1-expressing *M*. *smegmatis* was 72%, compared to 78% in non-expressing cells (Fig. 3B). In the case of NTD-expressing *M*. *smegmatis*, viability was a little bit higher 85%, compared to 80% in non-expressing cells (Fig. 3B). According to these results, the growth delay seen in MDP1-expressing *M*. *smegmatis* cannot be explained by the cell death caused by the experimental artifacts.

### MDP1 induces nucleoid compaction in an IDR-dependent manner

**Figure 4:**
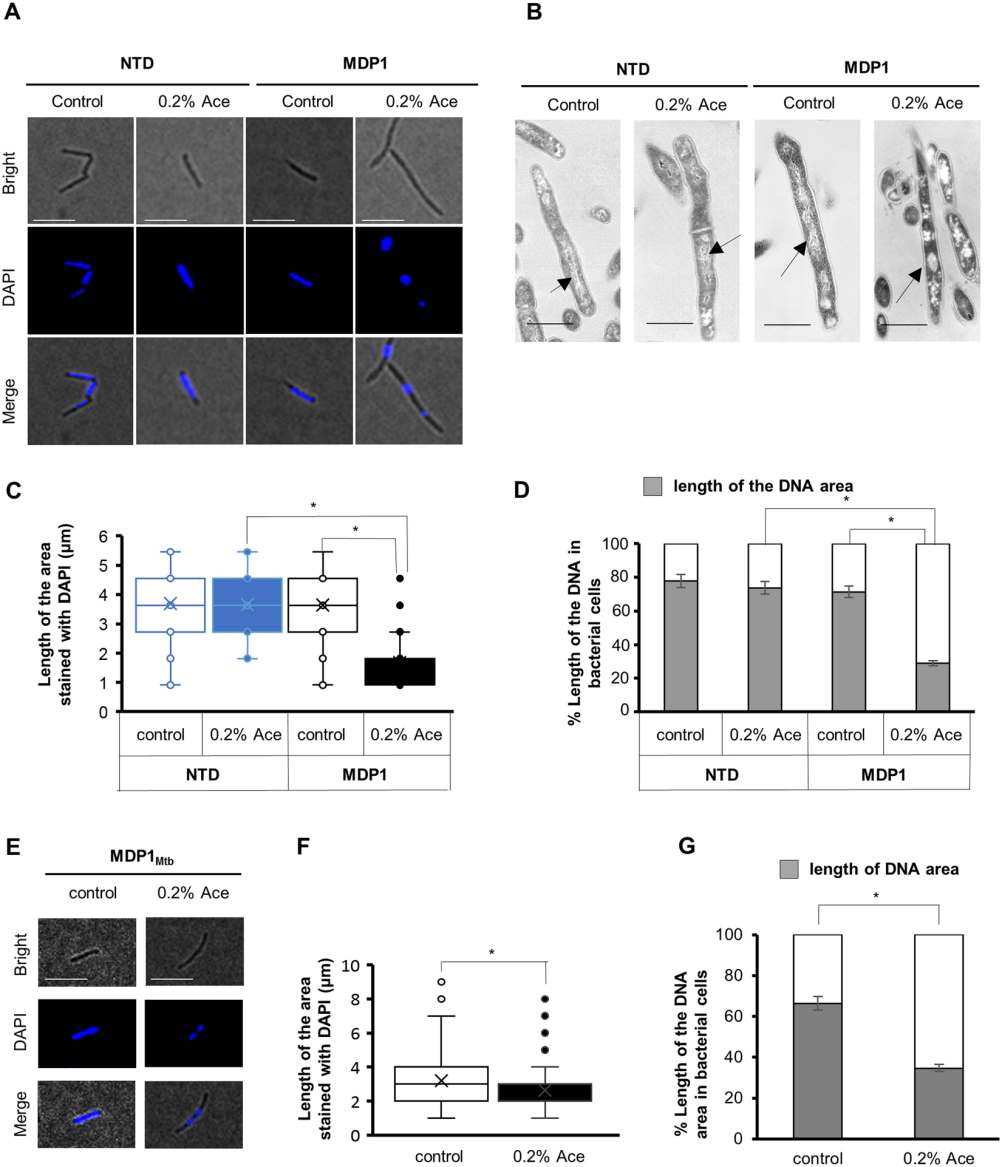
The MDP1 expression causes DNA compaction. (**A**) Fluorescence microscopic analysis of the cell and DNA morphology of AMI-*mdp1* (MDP1) and AMI-*NTD* (NTD). After 24 h incubation of bacteria in the presence or absence of 0.2% Ace (indicated as 0.2% Ace or control, respectively), bacterial cells were stained with DAPI and then the cell and DNA (nucleoid) morphology (blue) were analyzed under fluorescence microscope. Bright field (Bright), DAPI-stained (DAPI), and merged (Merge) images of represented cells are shown. Scale bars: 10 µm. (**B**) TEM analysis of bacterial structures of AMI-*mdp1* (MDP1) and AMI-*NTD* (NTD). Representative cell images in each group are shown. Arrows indicate nucleoid. Scale bars: 1 µm. (**C**) Distribution of the length of DAPI-stained DNA area of the cells detected in panel A (n ≥ 50). Open blue, AMI-*NTD* (−Ace, control); closed blue, AMI-*NTD* (+0.2% Ace); open black, AMI-*mdp1* (−Ace, control); and closed black, AMI-*mdp1* (+0.2% Ace). *P < 0.05. The result shown is a representative of at least three experiments. (**D**) Intracellular area occupied by DNA. Average lengths of DNA area under each condition of C were calculated. Percent intracellular area occupied by DNA is shown (gray). More than 100 cells were examined in each condition. Mean ± SD. *P < 0.05. (**E**) Fluorescence microscopic analysis of the cell and DNA morphology of AMI-*mdp1*_*Mtb*_, analyzed as described in A. Scale bars: 10 µm. (**F**) Distribution of the length of DAPI-stained DNA area of the cells detected in panel E (n > 100). Open black, AMI-*mdp1*_*Mtb*_ (−Ace, control); and closed black, AMI-*mdp1*_*Mtb*_ (+0.2% Ace). *P < 0.05. The result shown is representative of at least two experiments. (**G**) Intracellular area occupied by DNA. Average lengths of DNA area under each condition of F were calculated as shown in panel D (n > 100). Mean ± SD. *P < 0.05.

MDP1 is a kind of NAPs^14^ and its role in mycobacterial nucleoid condensation was suggested previously^21^. Because genome condensation is usually linked with growth arrest, we examined the role of the IDR in MDP1-dependent nucleoid condensation. After incubation of the strains in the presence or absence of 0.2% Ace, bacterial cells were stained with DAPI and analyzed using fluorescence microscopy. In the absence of MDP1, nucleoid DNA was spread into the cells (Fig. 4A, MDP1 [control] and NTD [control]). In contrast, marked DNA compaction was observed in the intact MDP1-expressing cells (Fig. 4A, MDP1 [0.2% Ace]). Interestingly, the cells expressing NTD showed similar DNA morphology to that of its control (Fig. 4A, NTD [0.2% Ace]). DNA compaction caused by MDP1 expression was also confirmed by TEM analysis (Fig. 4B).

Under fluorescence microscopy, we determined the lengths of bacterial cells (in polar axis) and of DAPI-stained DNA area (in polar axis). The average length of DNA area of MDP1-expressing *M*. *smegmatis* was 2.2 ± 0.15 µm (occupying 29% of the cell), compared to its control 3.7 ± 0.18 µm (71% of the cell) (Fig. 4C,D, *P* < 0.05). In contrast, the length of DNA area of NTD-expressing *M*. *smegmatis* cells (3.7 ± 0.11 µm, 74% of the cell) was similar to the control (Fig. 4C,D).

We also confirmed that similar DNA compaction was observed in MDP1_Mtb_-expressing *M*. *smegmatis* (Fig. 4E). After induction of MDP1_Mtb_ expression, the length of DNA area was 2.6 ± 0.062 µm (occupying 35% of the cell), compared to 3.2 ± 0.056 µm (66% of the cell) of its control (Fig. 4F,G, *P* < 0.05).

### The IDR-dependent induction of INH tolerance of *M*. *smegmatis*

INH is a first-line drug in TB chemotherapy and is efficent against mycobacteria at the logarithmic growth phase. However, mycobacteria at stationary to dormant phases become tolerant to INH^26,33^, which is a problematic charater of dormant mycobacteria causing the demand of long term chemotherapy. INH is a prodrug which is activated by catalase-peroxidase KatG in mycobacterial cells^34^. Our previous study showed that MDP1 expression is enhanced in stationary phase and confers tolerance to INH by down-regulating KatG expression^33^. Here we investigated whether C-terminal IDR is involved in this MDP1-induced INH tolerance.

**Figure 5:**
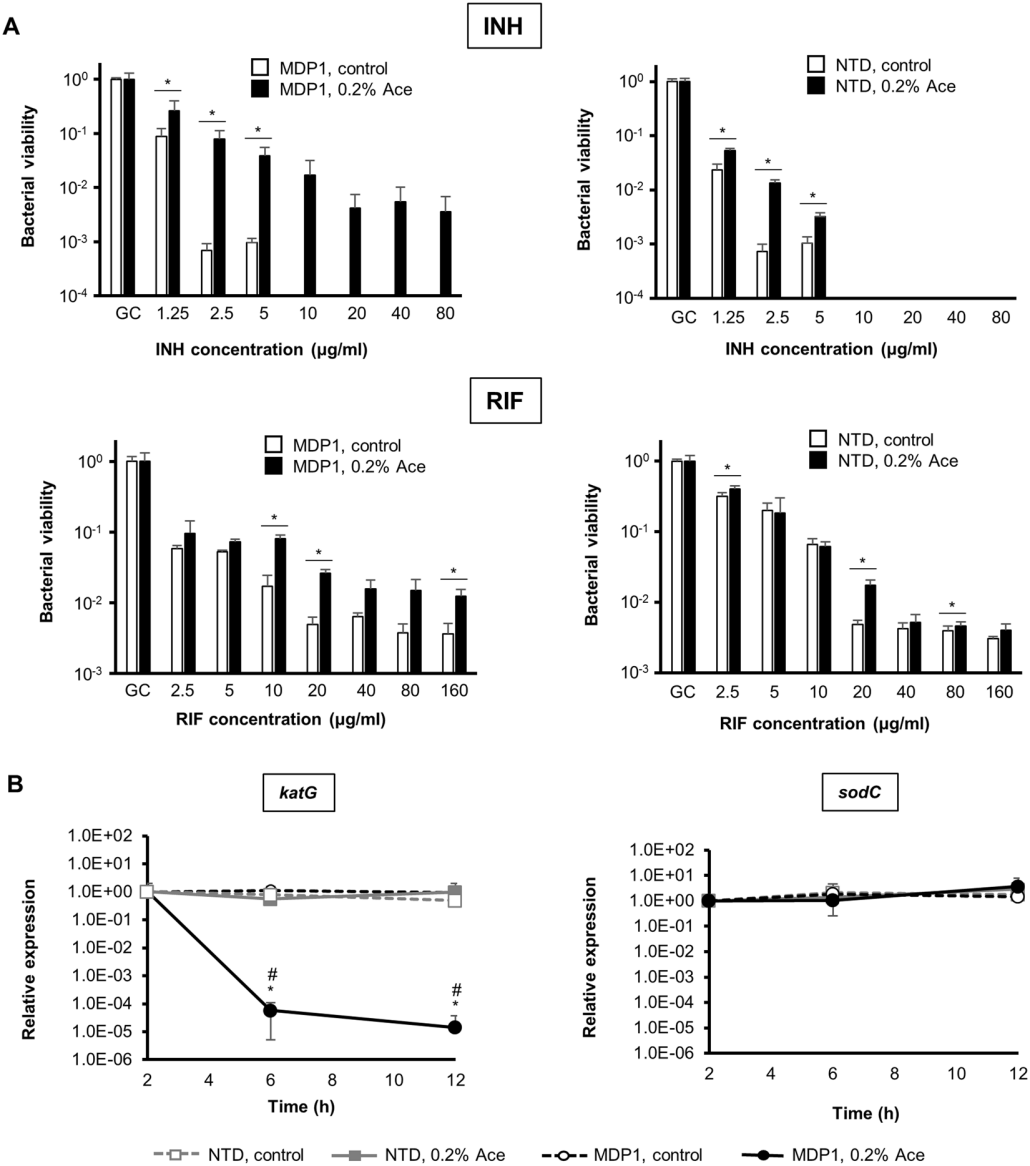
MDP1 but not NTD induced the bacterial tolerance to INH. (**A**) Drug susceptibility of AMI-*mdp1* and AMI-*NTD* in the presence and absence of 0.2% Ace. After 24 h incubation of bacteria in the presence or absence of 0.2% Ace, bacteria were exposed to the indicated concentrations of INH (upper panels) or RIF (lower panels) using broth microdilution method. After 24 h exposure, CFUs (CFU/ml) were determined and normalized with CFU of growth control (GC, no drug). Left and right panels represent AMI-*mdp1* (MDP1) and AMI-*NTD* (NTD) respectively. Closed and open bars represent data in the presence and abscence of 0.2% Ace respectively. Data are represented as mean ± SD (n = 3). *P < 0.05 between control and 0.2% Ace at each drug concentration. The result shown is representative of at least two experiments. (**B**) qRT-PCR analysis of *katG* (left panel) and *sodC* (right panel) expression. At 0 h, 0.2% Ace or a vehicle were added to AMI-*NTD* and AMI-*mdp1* cultures. At each time point, samples were harvested for analysis. Data was normalized by values at 2 h from each group. Solid black line with closed circle, dashed black line with open circle, solid gray line with closed square, and dashed gray line with open square represent AMI-*mdp1* + 0.2% Ace (MDP1, 0.2% Ace), AMI-*mdp1* without Ace (MDP1, control), AMI-*NTD* + 0.2% Ace (NTD, 0.2% Ace), and AMI-*NTD* without Ace (NTD, control) respectively. Data are represented as mean ± SD (n=3). * and ^#^ *P* < 0.05 compared to control and NTD (0.2% Ace), respectively, at each time point.

Without the induction of MDP1 and NTD expression, CFUs of both AMI-*mdp1* and AMI-*NTD* after exposure to INH at ≥10 μg/ml were under detectable level (<100 CFU/ml, Fig. 5A, INH). In contrast, after induction of MDP1 expression, viable cells were detected even after exposure to up to 80 µg/ml of INH (Fig. 5A, INH left panel). Furthermore, the reduction of viability of MDP1-expressing cells after treatment with 1.25–5 µg/ml INH was much smaller (4–26-fold reduction, compared to no drug control) than those of nonexpressing cells (11–1443-fold reduction, compared to no drug control). As for AMI-*NTD*, although non-expressing cells were more susceptible to INH at ≤5 µg/ml than NTD-expressing cells, the CFU of NTD-expressing cells after exposure to INH at≥10 μg/ml was under detectable level (<100 CFU/ml, Fig. 5A, INH right panel).

We further examined the KatG gene (*katG*) expression under the expression of MDP1 or NTD. After addition of Ace to each strain’s culture, time-course of the change of *katG* expression in the presence or absence of Ace was determined by quantitative real-time PCR (qRT-PCR). As expected, we observed the reduction of *katG* expression from 6 h after induction of intact MDP1 expression and this reduction was maintained for at least 12 h (Fig. 5B, *katG*). In contract, such reduction was not seen in its control or NTD-expressing bacteria (Fig. 5B, *katG*). As a control, we assessed the expression levels of superoxide dismutase C gene (*sodC*, MSMEG_0835) whose expression was not affect by MDP1^22^. Neither intact MDP1 nor NTD significantly altered *sodC* expression (Fig. 5B, *sodC*). Taken together, intact MDP1, but not NTD, suppresses *katG* transcription. This data suggests that MDP1 suppresses *katG* expression in a C-terminal IDR-dependent manner, resulting in the induction of IHN tolerance.

Rifampicin (RIF) is another front line TB drug and effective against dormant mycobacteria unlike INH^35,36^. In our previous report, knockout of genomic MDP1 gene from *M*. *smegmatis* did not alter bacterial susceptibility to RIF in the conventional broth microdilution assay^33^. Even in the presence of ≥10 µg/ml RIF which prevented visible growth of either wild-type strain or Δ*mdp1* previously^33^, both AMI-*mdp1* and AMI-*NTD* strains showed detectable CFUs in contrast to INH treatment (Fig. 5A, RIF). Under the same condition, MDP1-expressing *M*. *smegmatis* tended to survive more than its non-expressing control although the effect of MDP1 expression was not so remarkable, compared to that in INH susceptibility (Fig. 5A, RIF left panel). The effect of NTD expression on RIF susceptibility was also not remarkable (Fig. 5A, RIF right panels).

### Impact of MDP1 and NTD on syntheses of DNA and RNA in mycobacteria

**Figure 6:**
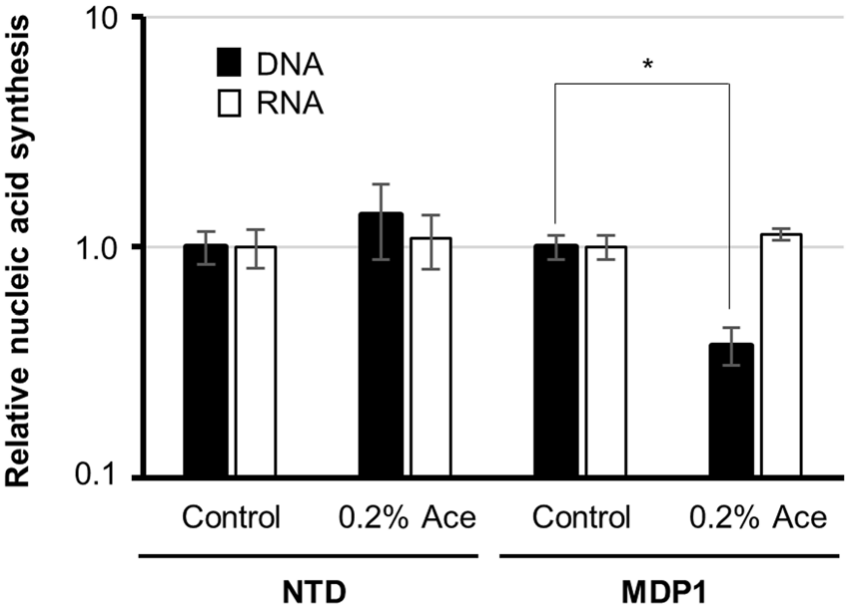
The effects of MDP1 and NTD expression on DNA and RNA synthesis. After incubation of AMI-*NTD* (NTD) and AMI-*mdp1* (MDP1) for 24 h in the presence or absence of 0.2% Ace, bacteria were diluted to 0.1 of A600 and then cultured for additional 24 h in the presence of [5, 6-^3^H] uracil. After extraction of nucleic acids as described in *Materials and Methods*, DNA and RNA syntheses were determined by the [5, 6-^3^H] uracil incorporation. Counts per minute (cpm) per A600 (cpm/A600) of each Ace-treated sample were normalized with those of untreated control and shown as relative nucleic acid synthesis. Closed and open symbols, DNA and RNA, respectively. Data are represented as mean ± SD (n = 3). *P < 0.05.

Recently we showed higher DNA synthesis (replication) in *Δmdp1*, compared to its wild-type and MDP1-complemented strains^22^. To determine whether the C-terminal IDR-dependent nucleoid compaction is correlated with MDP1-dependent suppression of DNA synthesis, we next measured the level of DNA (replication) and/or RNA (transcription) synthesis in the presence or absence of intact MDP1 or NTD. After induction of intact MDP1, two-fold decrease in DNA synthesis of AMI-*mdp1* was observed, compared to its control culture (Fig. 6). In contrast, AMI-*NTD* showed similar levels of DNA synthesis in the presence or absence of NTD (Fig. 6). This data suggests the relationship between the C-terminal IDR-dependent DNA condensation and suppression of DNA synthesis.

In contrast, RNA synthesis of either MDP1 or NTD expressing *M*. *smegmatis* was not significantly different from their controls (Fig. 6), suggesting that most of the RNA polymerase activity, which is the target of RIF, remained under MDP1 expression.

### MDP1 mediates DNA compaction in *M*. *smegmatis* cell

**Figure 7:**
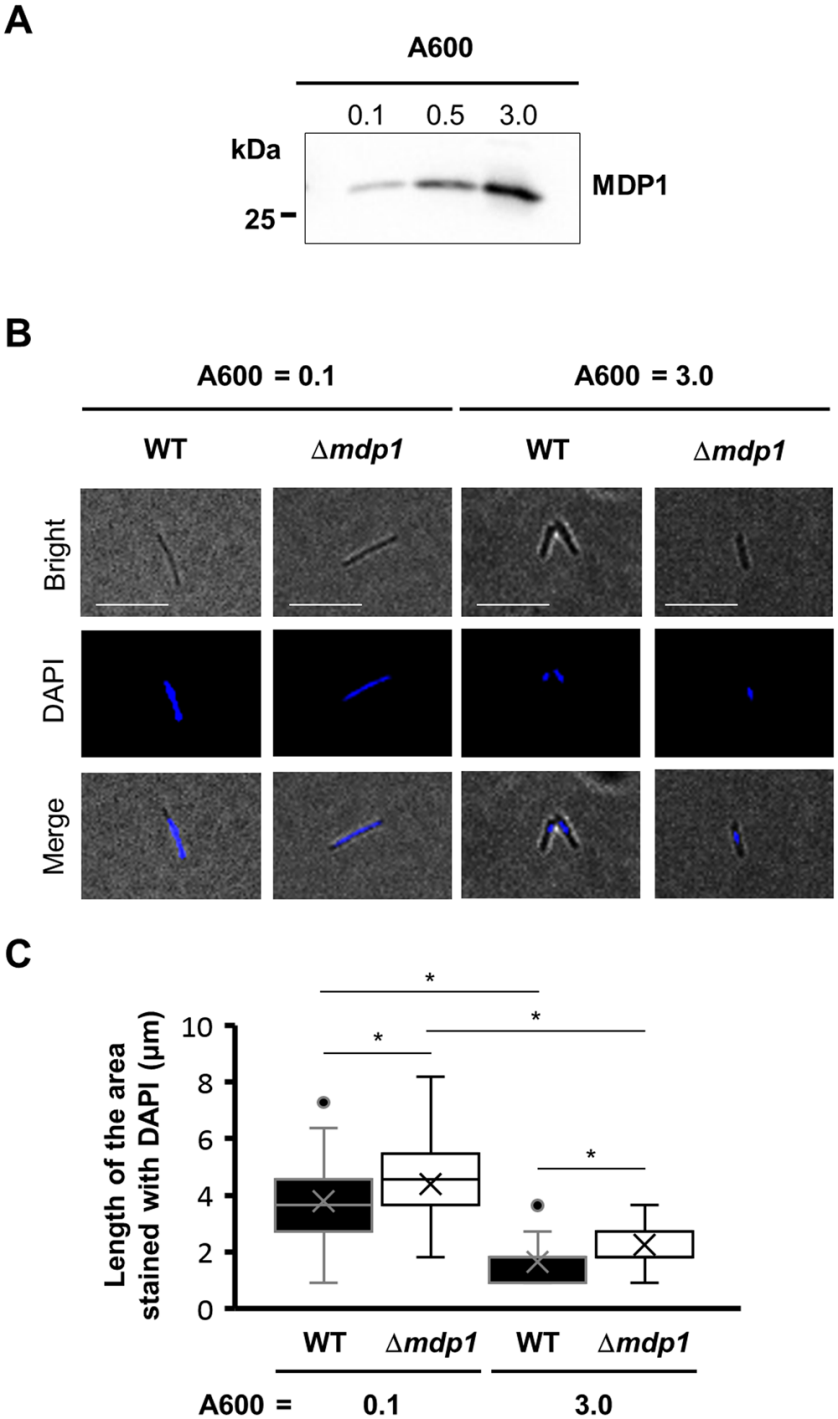
The analysis of the DNA morphology of wild-type and MDP1-deficient *M*. *smegmatis* during growth. (**A**) Western blot analysis of endogenous MDP1 in wild-type *M*. *smegmatis* mc^2^_155. *M*. *smegmatis* mc^2^_155 was harvested at the indicated A600 and MDP1 level was analyzed by western blotting. A full length western blot of this panel was also presented as Supplementary Figure S5. (**B**) Fluorescence microscopy of the DNA morphology of wild-type and Δ*mdp1*. Wild-type *M*. *smegmatis* mc^2^_155 (WT) and Δ*mdp1* harvested at the indicated A600 were stained with DAPI and visualised under a fluorescence microscope. Bright field images (Bright), DAPI-stained images (DAPI), and merged images (Merge) of representative cells are shown. Scale bars: 10 µm. (**C**) Distribution of the length of DNA area of WT and Δ*mdp1*. Sizes of DAPI-stained DNA spot (in polar axis) detected in panel B were further measured and plotted (n > 70). Closed and open symbols represent WT and Δ*mdp1* respectively. *P < 0.05.

The above data prompted us to examine the physiological role of endogenous MDP1 in mycobacterial cells’ DNA compaction. As we reported previously, endogenous MDP1 level in wild-type *M*. *smegmatis* mc^2^_155 increases from late logarithmic growth phase toward stationary phase (Fig. 7A)^22^. We compared the nucleoid morphology between Δ*mdp1* and wild-type strain in both logarithmic growth and staionary phases. As shown in Fig. 7B,C, similar nucleoid compaction was observed in both wild type *M*. *smegmatis* mc^2^_155 and Δ*mdp1*. However and most importantly, the wild-type strain always showed more compacted nucleoids at each A600 value, compared to that of Δ*mdp1* (Fig. 7C, *P* < 0.05). It is known that nucleoid compaction is caused by several revealed and unknown factors, such as, supercoiling of DNA by the DNA gyrase and topoisomarases, the molecular crowding of cytoplasmic space, and nucleoid-associated proteins. Our data demonstrate that MDP1 absolutely participates in the nucleoid compaction in *M*. *smegmatis* cell.

**Figure 8:**
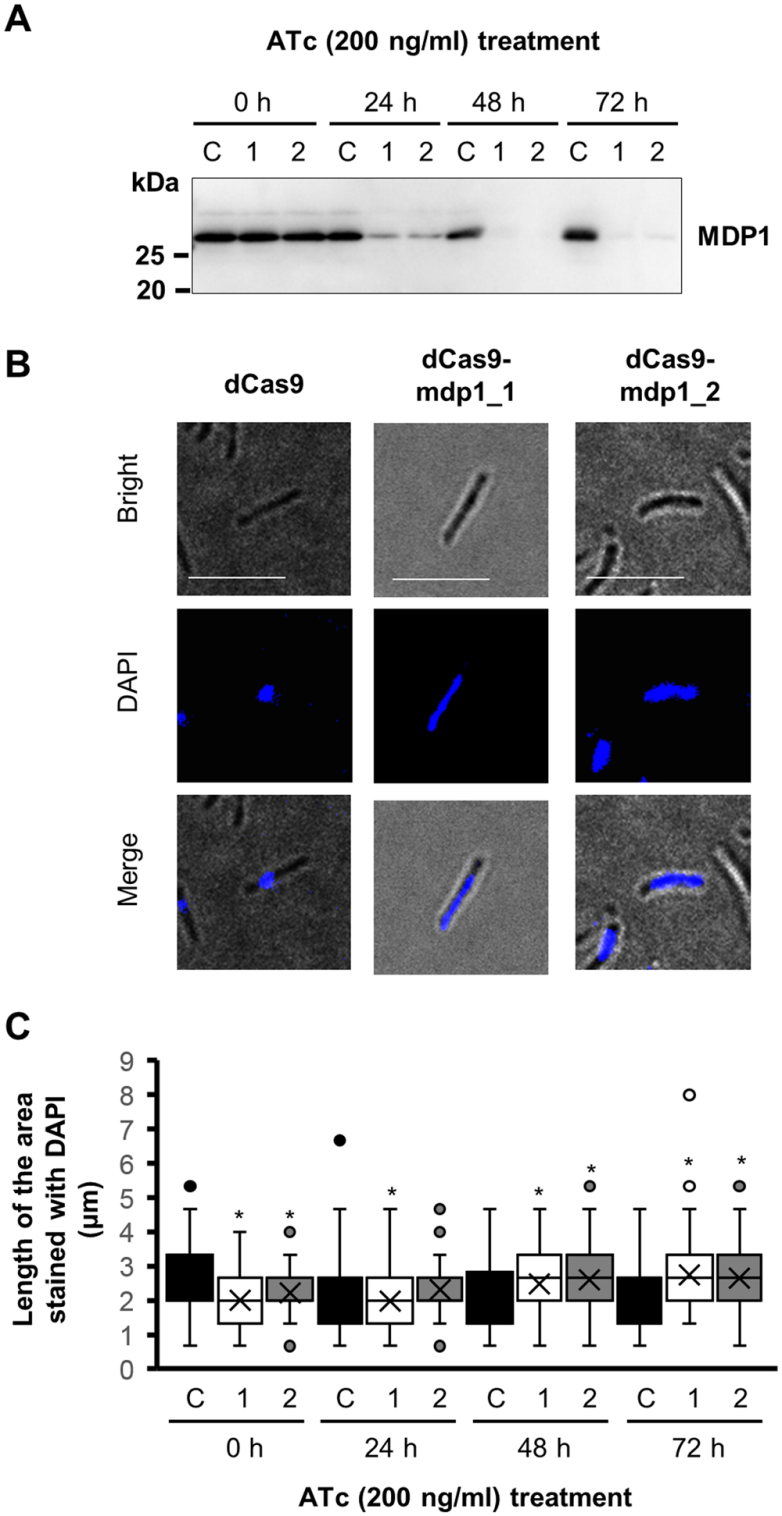
Altered DNA morphology by conditional reduction of MDP1 in *M*. *smegmatis*. (**A**) Western blot analysis of MDP1 protein in MDP1-conditional knock-down strains (dCas9-mdp1_1 and dCas9-mdp1_2) and a vector control (dCas9). dCas9-mdp1_1(1), dCas9-mdp1_2(2), and dCas9 (**C**) were cultured in the presence of 200 ng/ml ATc and harvested at the indicated time. MDP1 levels were analyzed by western blotting. A full length western blot of this panel was also presented as Supplementary Figure S6. (**B**) Fluorescence microscopy of the DNA morphology of MDP1-conditional knock-down *M*. *smegmatis*. dCas9-mdp1_1, dCas9-mdp1_2, and dCas9 were cultured and harvested as described in panel A. DNA was stained with DAPI and analyzed under a fluorescence microscope. Bright field images (Bright), DAPI-stained images (DAPI), and merged images (Merge) of representative cells are shown. Scale bars: 5 µm. (**C**) Distribution of the length of DNA area of MDP1-conditional knock-down *M*. *smegmatis*. Sizes of DAPI-stained DNA spot (in polar axis) analyzed in B were further measured and plotted (n > 90). C (black), dCas9: 1 (white), dCas9-mdp1_1; and 2 (gray), dCas9-mdp1_2. *P < 0.05 compared to dCas9 at each time point.

In order to know the precise role of MDP1 in genome condensation at stationary phase, we constructed MDP1-conditional knockdown strains of *M*. *smegmatis* mc^2^_155 using TetR/O-based CRISPR/dCas9 interference system^37^. Incubation of MDP1-conditional knockdown strains (dCas9-mdp1_1 and dCas9-mdp1_2) with 200 ng/ml anhydrotetracycline (ATc) induced the depletion of cellular MDP1 protein at stationary growth phases (48 and 72 h later in Fig. 8A). After 48 h incubation of dCas9-mdp1_1 and dCas9-mdp1_2 with ATc, the MDP1 protein was under detectable level by western blot (Fig. 8A). At the same time point, we investigated the change of nucleoid DNA morphology and revealed that, after 48 h incubation with ATc, dCas9-mdp1_1 and dCas9-mdp1_2 showed more spread DNA, compared to its control (Fig. 8B,C, *P* < 0.05). Taken together, these data indicate the essential role of endogenous MDP1 in the DNA condensation process from logarithmic growth phase towards stationary phase.

## Discussion

Mycobacteria express a unique histone-like protein, MDP1, which consists of an N-terminal HU-like region and a C-terminal IDR. In this study, we observed that IDR-deleted MDP1 loses its functions, such as genome compaction (Fig. 4), growth arrest (Fig. 2), and tolerance to INH (Fig. 5). This demonstrates that IDR is essential for MDP1-functions.

One possible explanation of this phenomenon is the reduced DNA-binding activity of MDP1 caused by the loss of IDR. This is supported by the previous observation in which NTD alone showed only limited affinity to short strand DNA in cell-free assay, compared to intact MDP1^21,23,38^. The structural cooperation of NTD and IDR may be necessary to fold mycobacterial DNA into higher order conformations. This kind of functional relationship between folded and unfolded domains is similar to that observed in eukaryotic histone H1, that is, the truncation of C-terminal IDR also abolished stable DNA binding of histone H1^9^.

Another possible explanation is that IDR-deleted MDP1 loses binding capacities with regulatory proteins that interact with MDP1-IDR. Importantly, exposed IDRs enable multiple interactions along their polypeptide chains. It is known that the interaction between IDR and partner molecules stabilizes IDR and in turn express the function. Such an interaction sometimes makes IDR hubs in the functional protein complexes^39^. The loss of such possible interactions and hubs dependent on IDR may explain the loss of function of NTD. Identification of MDP1-IDR binding proteins may be the next important step to elucidate total function of MDP1.

Our data clearly show that MDP1 mediates compaction of mycobacterial nucleoid (Figs 7 and 8). This compaction is dependent on the C-terminal IDR (Fig. 4) that resembles the eukaryotic linker histone H1. As for histone H1, the C-terminal IDR tail contains predominantly Lys (38–42%), Ala (17–34%), and Pro (12–14%)^40^. The central globular domain of histone H1 binds to the nucleosome whereas the C-terminal IDR tail binds to the linker DNA. The C-terminal IDR tail induces apposed stem/loop motif in the linker DNA and stabilizes folded or oligomeric conformation of chromatin fibers^7,41^. Lu & Hansen distinguished C-terminal IDR tail of histone H1 into 24/25-amino acid subdomains and revealed the functions (such as alteration of chromatin fiber conformation) in each subdomains^7^. Amino acid composition and position, rather than primary sequence, of subdomains determine the ability of each subdomain^42^. This amino acid composition seems important to form short α-helices in these subdomains in response to DNA binding.

In case of MDP1, C-terminal IDRs are diverged from histone H1 C-terminal IDR in terms of primary sequence, but similar in terms of amino acid composition (e.g., 33% of Lys, 42% of Ala, and 10% of Pro in *M*. *smegmatis* MDP1; 30% of Lys, 35% of Ala, and 8% of Pro in *M*. *tuberculosis* MDP1_Mtb_; Fig. 1)^14^. Therefore, it is possible that DNA-binding by MDP1 induces an α-helical structure in the C-terminal IDR. Indeed, a few α-helices were predicted in C-terminal half by secondary structure prediction of *M*. *smegmatis* MDP1 and *M*. *tuberculosis* MDP1_Mtb_ (Supplementary Fig. 1). Our data also highlights the significance of amino acid composition of C-terminal IDR in nucleoid compaction and growth delay since *M*. *smegmatis* MDP1 and *M*. *tuberculosis* MDP1_Mtb_ induced similar phenotypes (Figs 2 and 4). Taken these factors together, in MDP1, folding of NTD and DNA-binding-induced folding of C-terminal IDR may cooperatively induce stable binding to DNA and high order condensation of the nucleoid.

The nucleoid of *M*. *smegmatis* is more condensed in the stationary phase than in the logarithmic growth phase and MDP1 is involved in this process (Figs 7 and 8). Nucleoid condensations are seen in inactivated forms of other bacteria, e.g., the elementary body of *Chlamydia* and the stationary phase of *E*. *coli*^43,44^. Interestingly, histone-like proteins play a pivotal role in these processes in such organisms, implying a common significance of histone-like proteins in nucleoid condensation. Depending on the multiplication of the cells, toxic metabolites accumulated in growth-arrested bacteria as a result of loss of dilution effects. Thus, to prevent toxic metabolites-induced damages a compact nucleoid may be feasible to maintain the genome integrity during bacterial persistence.

Nucleoid condensation is generally related with the reduction of transcription as seen in heterochromatin in eukaryotes and X-chromosome inactivation in mammal females^45–47^. In this study, *katG* transcription is remarkably suppressed by MDP1 expression, but not NTD (Fig. 5B), which in turn, afforded INH-tolerance (Fig. 5A). Recently, we also reported that, in the stationary phase, MDP1 suppresses 22 protein expression with more than 3 folds, which include proteins involved in mycobacterial replication and metabolism for example KatG^22^. In addition, under the CFU-based method, MDP1 expression seems somewhat protective against RIF, an inhibitor of transcription (Fig. 5A). However, unexpectedly MDP1 did not significantly affect total RNA synthesis of *M*. *smegmatis* (Fig. 6). It can be considered that silencing of the entire gene expression by genome compaction may cause death of bacteria since bacteria, including mycobacteria, are single cell organisms. For instance, H-NS is known as a global gene repressor that suppresses a wide variety of genes located around its binding regions by bridging the adjacent binding regions^30,31^ and its overexpression causes lethal phenotype^32^. In contrast and interestingly, MDP1 expression just induces growth arrest but does not result in bacterial death (Fig. 3). Taken together, genome condensation induced by up-regulated MDP1 may be a feasible mechanism for mycobacteria to adapt to a persistent state after the stationary phase as such, regulation of gene expression via nucleoid condensation is the next important issue for understanding mycobacterial persistence.

To our knowledge, this is the first report suggesting the significant role of IDR in organizing the genome architecture in bacteria. This study provides the fundamental knowledge for understanding IDR function in bacteria.

## Methods

### Bacterial strains, culture media, and general reagents

Δ*mdp1* was kindly provided by Dr. John L. Dahl (University of Minnesota Duluth)^26^. *E*. *coli* DH5α strain was used for all gene manipulation in this study. All *M*. *smegmatis* strains were grown in Middlebrook 7H9 broth (BD, Franklin Lakes, NJ) supplemented with 0.2% (v/v) glycerol, 0.05% (v/v) Tween 80 (MP Biomedicals, Santa Ana, CA), and 10% ADC enrichment (5% bovine serum albumin [Wako Pure Chemical Industries, Osaka, Japan], 0.81% NaCl, and 2% D-glucose) (7H9-ADC broth) or on Mycobacteria 7H11 agar (BD) supplemented with 0.5% (v/v) glycerol and 10% OADC enrichment (ADC enrichment supplemented with 0.06% [v/v] oleic acid) (7H11-OADC agar). Appropriate antibiotics were also added to the media to maintain the specific genotypes of each strain. All *E*. *coli* strains were cultured in LB broth or on LB agar (both from Sigma-Aldrich, St. Louis, MO). Hygromycin B (Hyg), kanamycin (Km), and RIF were purchased from Wako Pure Chemical Industries (Osaka, Japan). INH and Ace were purchased from Sigma Aldrich. ATc was purchased from Cayman Chemical (Ann Arbor, MI).

### Construction of *M*. *smegmatis* mc^2^_155 strains inducibly expressing intact MDP1 or NTD

Sequences encoding His-tagged intact MDP1 (*mdp1 [MSMEG_2389]-His*) and NTD (*NTD-His*) of *M*. *smegmatis* mc^2^_155, and His-tagged MDP1_Mtb_ of *M*. *tuberculosis* H37Rv (*mdp1*_*Mtb*_ *[Rv2986c]-His*) were amplified by PCR using the primer sets listed in Supplementary Table S1. Amplified DNA fragments were excised with HindIII and KpnI and then inserted between HindIII and KpnI sites of pSO246 (Km^R^)^48^. The DNA cassette (kindly provided by Dr. Naoya Ohara, Okayama University) which involves the loci from acetamidase (MSMEG_5335) promoter region to MSMEG_5339^49,50^ was originally amplified from *M*. *smegmatis* genomic DNA using a primer set listed in Supplementary Table S1 and cloned in pCRII-TOPO (Km^R^). DNA cassette of AMI was excised with BamHI and NdeI, and then inserted between BamHI and NdeI sites (at the first Met codon of *mdp1-His*, *NTD-His*, and *mdp1*_*Mtb*_*-His*) of the plasmids (finally designated as pSO246-AMI-*mdp1*, -*NTD*, and -*mdp1*_*Mtb*_, respectively). Δ*mdp1* (Hyg^R^) was then transformed by constructed plasmids and inducibly expressing clones (designated as AMI-*mdp1*, AMI-*NTD*, and AMI-*mdp1*_*Mtb*_) were selected on the agar plates containing 50 μg/ml Hyg and 10 μg/ml Km. For induction of MDP1 proteins, Ace was supplemented to bacterial culture at final concentration indicated in each experiment.

### Gene knockdown by CRISPR interference

pRH2502, a vector expressing an enzymatically inactive Cas9 (dCas9), and pRH2521, a vector expressing guide RNAs (sgRNAs), were kindly gifted by Dr. Robert N. Husson^37^. Both dCas9 and sgRNA are expressed from TetR-regulated promoters (*uvtetO* and *P*_*myc1-tetO*_, respectively). For knockdown of *mdp1* in *M*. *smegmatis*, two sgRNAs were designed for targeting non-template strand of *mdp1* (#1; AAATGGGCACCGATCGTCGG, #2; CCGCGGTGGAGAACGTCGTC). To minimize off-target effect, we confirmed that there was no gene which had similar sequence to sgRNA with less than 5 mismatches by a BLAST search. Each designed oligonucleotide for sgRNA expression was inserted into pRH2521 (designated pRH2521-*mdp1*#1 and pRH2521-*mdp1*#2). *M*. *smegmatis* cells, which were already transformed by pRH2502 (for dCas9 expression), were transformed by these vectors or pRH2521 and then strains were selected on agar plates containing 50 μg/ml Hyg and 10 μg/ml Km (designated dCas9-mdp1_1, dCas9-mdp1_2, and dCas9). For induction of dCas9 and sgRNA expression (knockdown of *mdp1*), ATc was supplemented to bacterial culture at a final concentration of 200 ng/ml.

### Bacterial culture

*M*. *smegmatis* mc^2^_155, grown in the middle of a logarithmic phase, was inoculated in 7H9-ADC broth at 0.005 of A600 and then cultured at 37 °C with gentle stirring by the magnetic stirring bar at 60 rpm. The aliquots of the culture were harvested at the time points, indicated in each experiment, and used for the analyses.

AMI-*mdp1*, AMI-*NTD*, and AMI-*mdp1*_*Mtb*_ were pre-cultured in 7H9-ADC broth supplemented with 50 μg/ml Hyg and 10 μg/ml Km in the absence of Ace at 37 °C with gentle stirring (60 rpm) using a magnetic stirring bar till around 0.5 of A600 to maintain the logarithmic growth of bacteria. To determine the optimal conditions to induce protein expression, protein synthesis of the constructed strains were induced at 0.5 of A600 by the addition of Ace for a final concentration indicated in Figure. Cells were harvested at 24–72 h after incubation and protein expression levels were analysed by western blotting.

As for the analysis of growth kinetics, precultures of AMI-*mdp1*, AMI-*NTD*, and AMI-*mdp1*_*Mtb*_ were diluted to 0.005 of A600 with fresh media containing 0.2% Ace or not and then cultured at 37 °C with gentle stirring by a magnetic stirring bar at 60 rpm. At the indicated time points in each experiment, bacterial cells were harvested to determine A600 values, colony-forming units (CFUs) and for western blotting.

As for microscopic analyses, pre-cultures of the strains were diluted to 0.1 of A600 with fresh media containing 0.2% Ace or not. Cells were harvested 24 h after addition of Ace and used for analysis.

dCas9-mdp1_1, dCas9-mdp1_2, and dCas9 were precultured in 7H9-ADC broth supplemented with 50 μg/ml Hyg and 10 μg/ml Km in the absence of ATc at 37 °C with gentle stirring by the magnetic bar at 60 rpm till around 0.5 of A600 to maintain the logarithmic growth of bacteria. To deplete cellular MDP1 protein, precultures were diluted to 0.1 of A600 with fresh medium containing ATc (final 200 ng/ml). Bacterial cells were harvested at 24–72 h after incubation and used for analysis.

### CFU measurement

CFUs of each culture were determined as follows. Ten-fold dilutions of each culture from 10^−1^ to 10^−7^ were prepared and then plated in triplicate on 7H11-OADC agar. The plates were cultured at 37 °C for 3–4 days. The number of colonies were then counted and CFUs (CFU/ml) were calculated.

### Determination of MDP1 protein expression levels by western blot analysis

Bacterial cells harvested above were washed three times with ice-cold water by centrifugation at 10,000 rpm and 4 °C. To eliminate lipid compounds, the cell pellet was treated with 100% acetone for 10 min at room temperature (RT) and then air-dried. Glass beads (1 mm diameter) and water were added to the cell pellet and then the cells were disrupted by BeadSmash BS-12R (Wakenyaku, Kyoto, Japan) at 5,500 rpm, 4 °C for 30 sec three times with 30 sec interval between operations. Protein concentration in the bacterial whole cell lysate was measured by Pierce^TM^ BCA Protein Assay kit (Thermo Fisher Scientific, Waltham MA). After treatment of the samples with Laemmli sample buffer at 95 °C for 5 min, 10 μg of proteins were electrophoresed on a sodium dodecyl sulphate (SDS)-polyacrylamide gel and transferred to Immobilon-P^SQ^ membrane (Merk Millipore, Billerica, MA). After blocking the membrane with skim milk, endogenous MDP1 protein was detected with mouse monoclonal anti-MDP1 antibody^33^. Primary antibodies were further detected with horse radish peroxidase (HRP)-conjugated rabbit anti-mouse immunoglobulins. On the other hand, His-tagged MDP1 proteins were detected with rabbit polyclonal anti-6-His antibody (Bethyl Laboratories, Inc., Montgomery, TX) and HRP-conjugated donkey anti-rabbit immunoglobulin G (H + L) antibody (Jackson ImmunoResearch Laboratories, Inc., West Grove, PA). After treatment of the membrane with Amersham^TM^ ECL^TM^ Prime Western Blot Detection Reagent (GE Healthcare) according to the manufacturer’s instruction, bands were visualised using ATTO Ez Capture MG system (ATTO, Tokyo, Japan).

As for densitometric analysis to quantify His-tagged MDP1 and NTD on the membrane, dilutions of recombinant His-tagged MDP1 at various known concentrations were electrophoresed along with whole cell lysate samples. After detection, band densities of MDP1 and NTD proteins were analyzed using CS Analyzer 3.0 software (ATTO). A standard curve was prepared from the recombinant His-tagged MDP1 protein data. The concentrations of MDP1 or NTD in 10 µg of total protein of whole cell lysate samples were calculated using the standard curve.

### Viability assay of the *M*. *smegmatis* strains by fluorescence microscopy

The viability of mycobacterial cells was measured using LIVE/DEAD *Bac*Light Bacterial Viability kit (Thermo Fisher Scientific). *M*. *smegmatis* strains were harvested by centrifugation at 800 *g* for 10 min and washed once with presterile water. Cells were then stained with SYTO9 and PI at RT for 20 min using Live/Dead *Bac*Light Bacterial viability kit according to the manufacturer’s instruction and then mounted on a slide. Fluorescence images were visualised with a x100 oil immersion objective lens on an All-in-one BZ-X700 Series fluorescence microscope (Keyence, Osaka, Japan). Numerous randomly selected microscopic fields (at least 150 bacterial cells) from slides of triplicate cultures were examined to count the numbers of viable (stained with SYTO 9) and dead (stained with PI) cells. The percentage of viable cells was determined.

### Fluorescence microscopic analysis of DNA morphology

Bacterial cells harvested by centrifugation at 800 *g* for 10 min, were washed with PBS and then treated with 10 µg/ml DAPI (Thermo Fisher Scientific) at RT for 20 min under the protection from light. Stained cells were washed with PBS and mounted on a slide. The cell and nucleoid morphologies were visualised with a x100 oil immersion objective on an All-in-one BZ-X700 Series fluorescence microscope. Lengths of DNA area (blue spots) in the polar axis direction of bacterial cells were measured. Slides of triplicate cultures (at least 60 bacterial cells per slide) from numerous randomly selected microscopic fields were examined.

### TEM analysis

One ml of bacterial suspension was centrifuged at 800 *g* for 10 min and harvested cells then washed once with water. The cell pellet was fixed with 2.5% glutaraldehyde in 0.1 M phosphate buffer of pH 7.4 overnight at 4 °C. The fixed cells were then post-fixed for 1 h in 1% osmium tetroxide in 0.1 M phosphate buffer of pH 7.4. Cells were then dehydrated with graded concentrations of ethanol and then embedded in epoxy resin (Quetol-651, Nissin EM, Tokyo, Japan). The resin blocks of the samples were thinly sectioned by ultra microtome Ultracut-N (Reichert-Nissei, Tokyo, Japan) to obtain ultrathin sections of 90 nm. Sections were then stained with uranyl acetate and lead citrate. These prepared samples were then visualized using Hitachi transmission electron microscope H-600A (Hitachi, Tokyo, Japan).

### Antibiotic susceptibility test

Two-fold dilutions of the antibiotics at indicated concentration range were prepared with 7H9-ADC broth containing 0.2% Ace or not and dispensed in a 96-well microplate. Bacterial cells grown in the presence or absence of 0.2% Ace at 37 °C for 24 h, were inoculated to each well at 0.01 of A600 and cultured at 37 °C. After 24 h antibiotics were removed by centrifugation at 5,000 rpm for 10 min. Cells were further washed with sterile water and CFU (/mL) was determined.

### Quantitative real-time PCR assay (qRT-PCR)

Total RNA was extracted from bacterial cells using TRIzol reagent (Thermo Fisher Scientific) and Direct-zol^TM^ RNA MiniPrep kit (Zymo Research, Irvine, CA) according to manufacturer’s instructions, with some modifications. Briefly, bacterial cells grown in the presence or absence of 0.2% Ace as described above, were harvested by centrifugation at 9,000 *g*, 4 °C for 10 min. Cells were re-suspended in 1 ml TRIzol reagent and disrupted using glass beads (0.1 mm diameter) in a BeadSmash BS-12R as described before. The samples were frozen at −80 °C at least overnight. After thawing, aqueous fraction was recovered by centrifugation at 15,000 *g*. Total RNA was isolated from the aqueous fraction by isopropanol precipitation. Isolated total RNA was further purified using Direct-zol^TM^ RNA MiniPrep kit (Zymo Research, Irvine, CA). After determining RNA concentration and quality, cDNA was synthesised using the ReverTraAce qPCR RT Master Mix with gDNA Remover (Toyobo, Osaka, Japan) according to the manufacturer’s instructions. Reaction mixtures of qRT-PCR were prepared using THUNDERBIRD SYBR qPCR MIX (Toyobo) according to the manufacturer’s instructions. The sequences of the primer sets used in this study are shown in the Supplementary Table S1. The qRT-PCR reactions were performed using CFX Connect Real Time System (Bio-Rad Laboratories, Hercules, CA). Relative gene expression was determined by a calculated threshold cycle (CT) and data was normalised against *sigA* and 16 S rRNA as internal standards.

### RNA and DNA synthesis measurement by [5, 6-^3^H] uracil incorporation

RNA and DNA synthesis was determined as described before, with some modifications^51^. Bacterial cells grown in the presence or absence of 0.2% Ace as described above were diluted to 0.1 of A600. Sterile [5, 6-^3^H] uracil (final 37 kBq/ml) was added to the diluted cultures and incorporated into the cells for 1 h at 37 °C. Samples were then divided into two equal aliquots. One was used to determine the incorporation of [5, 6-^3^H] uracil to total nucleic acids and the other was incubated in 0.3 N KOH at 37 °C for 24 h to degrade RNA (radioactivity in total DNA). Bacterial cells in the culture were trapped on the 0.45 µm filter membrane (Merk Millipore) and washed with 20 ml water. Filter membranes were dissolved in 2-ethoxy ethanol for 2 hr at RT and then mixed with Ecoscint A scintillation cocktail before counting the radioactivity in a scintillation counter (Aloka liquid scintillation counter, Hitachi). As a background of radioactivity, a sterile medium was used. Radioactivity incorporated in total RNA was calculated by the subtraction of radioactivity in total DNA from that in total nucleic acids. The RNA and DNA synthesis were represented as a ratio of counts per minutes (cpm) per A600.

### Statistics

Data was analyzed using one-way ANOVA with post-hoc Tukey HSD test or Games-Howell post-hoc test, Wilcoxon/Kruskal-Wallis non-parametric test, and Student *t*-test. Differences were considered significant when the *P*-value was <0.05.

## Electronic supplementary material

Supplementary Information
