# Supplementary material for: C-terminal intrinsically disordered region-dependent organization of the mycobacterial genome by a histone-like protein
Source: Sci Rep. 2018 May 29;8:8197. doi: 10.1038/s41598-018-26463-9 (PMC5974015; doi:10.1038/s41598-018-26463-9)
Supplement: Supplementary file 1 — Supplementary Information [file 41598_2018_26463_MOESM1_ESM.pdf]

C-terminal intrinsically disordered region-dependent organization of the mycobacterial genome by a histone-like protein

Anna Savitskaya[1,3], Akihito Nishiyama[1]*, Takehiro Yamaguchi[1,4], Yoshitaka Tateishi[1], Yuriko Ozeki[1], Masaaki Nameta[2], Tomohiro Kon[1], Shaban A. Kaboso[1], Naoya Ohara[5], Olga V. Peryanova[3], and Sohkichi Matsumoto[1]*

Supplementary Table

Supplementary Table S1. Primer sequences used in this study.

Gene	Primer sequence		
**Gene cloning**			
mdp1-His6	Forward	5'-TTT AAG CTT GTT CAT ATG AAC AAA GCG GAG CTC ATC GAC-3'	This study
	Reverse	5'-GGT GGT ACC TTA GTG GTG GTG GTG GTG GTG TCG ACA CCT GCG GCC CTT CTT GGC CGG GG-3'	This study
NTD-His6	Forward	5'-TTT AAG CTT GTT CAT ATG AAC AAA GCG GAG CTC ATC GAC-3'	This study
	Reverse	5'-GGT GGT ACC TTA GTG GTG GTG GTG GTG GTG ACC ATC GGC CGG GAG CTT CTG TG-3'	This study
mdp1$_{Mtb}$-His6	Forward	5'-TTT AAG CTT GTT CAT ATG AAC AAA GCA GAG CTC ATT GAC-3'	This study
	Reverse	5'-GGT GGT ACC TTA GTG GTG GTG GTG GTG GTG TCG ACA TTT GCG ACC CCG CC-3'	This study
AMI	Forward	5'-GGA TCC CGA GTA CGG CGC CCT GCT GAC-3'	This study
	Reverse	5'-GCA TAT GGA CTC CCT TTC TCT TAT CGG GTG-3'	This study
**qRT-PCR**			
sigA	Forward	5'-CGT TCC TCG ACC TCA TCC A-3'	[1]
	Reverse	5'-GCC CTT GGT GTA GTC GAA CTT C-3'	[1]
16S rRNA	Forward	5'-TTC AAA GCC GGT CTC AGT TC-3'	[1]
	Reverse	5'-CGT TGC TGA TCT GCG ATT AC-3'	[1]
katG	Forward	5'-TTG TGT TCG GGT CAC ATT CG-3'	[1]
	Reverse	5'-TTT TGA CGA TCG GTG ACC AG-3'	[1]
sodC	Forward	5'-CAC GAG AAG GCC GAC AAC TT-3'	[1]
	Reverse	5'-GTG GCC ATC GTC GTC TGA TC-3'	[1]

Supplementary Figures

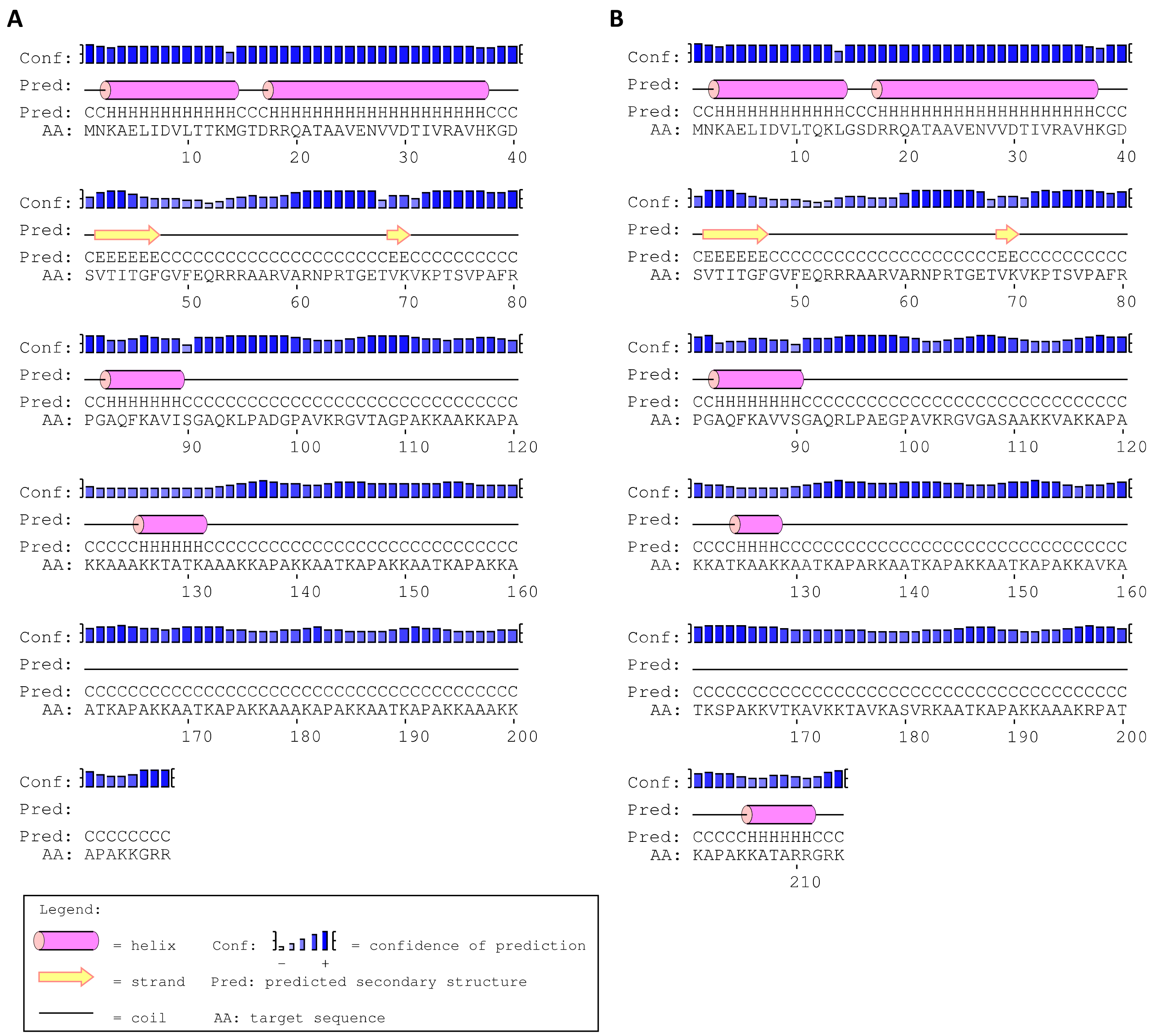

Supplementary Figure S1. Secondary structure prediction of MDP1 proteins.

Secondary structures of *M. smegmatis* MDP1 (MSMEG2389; **A**) and MDP1$_{Mtb}$ (Rv2986c; **B**) were predicted using PSIPRED (http://bioinf.cs.ucl.ac.uk/psipred/)[2]. Secondary structures (α helices and β strands) and confidence of prediction are indicated in each panel. Predicted structures in NTD (1 – 99 amino acid region) of both proteins are largely corresponding to those revealed by the crystal structure of NTD in MDP1$_{Mtb}$ (Fig. 1)[3].

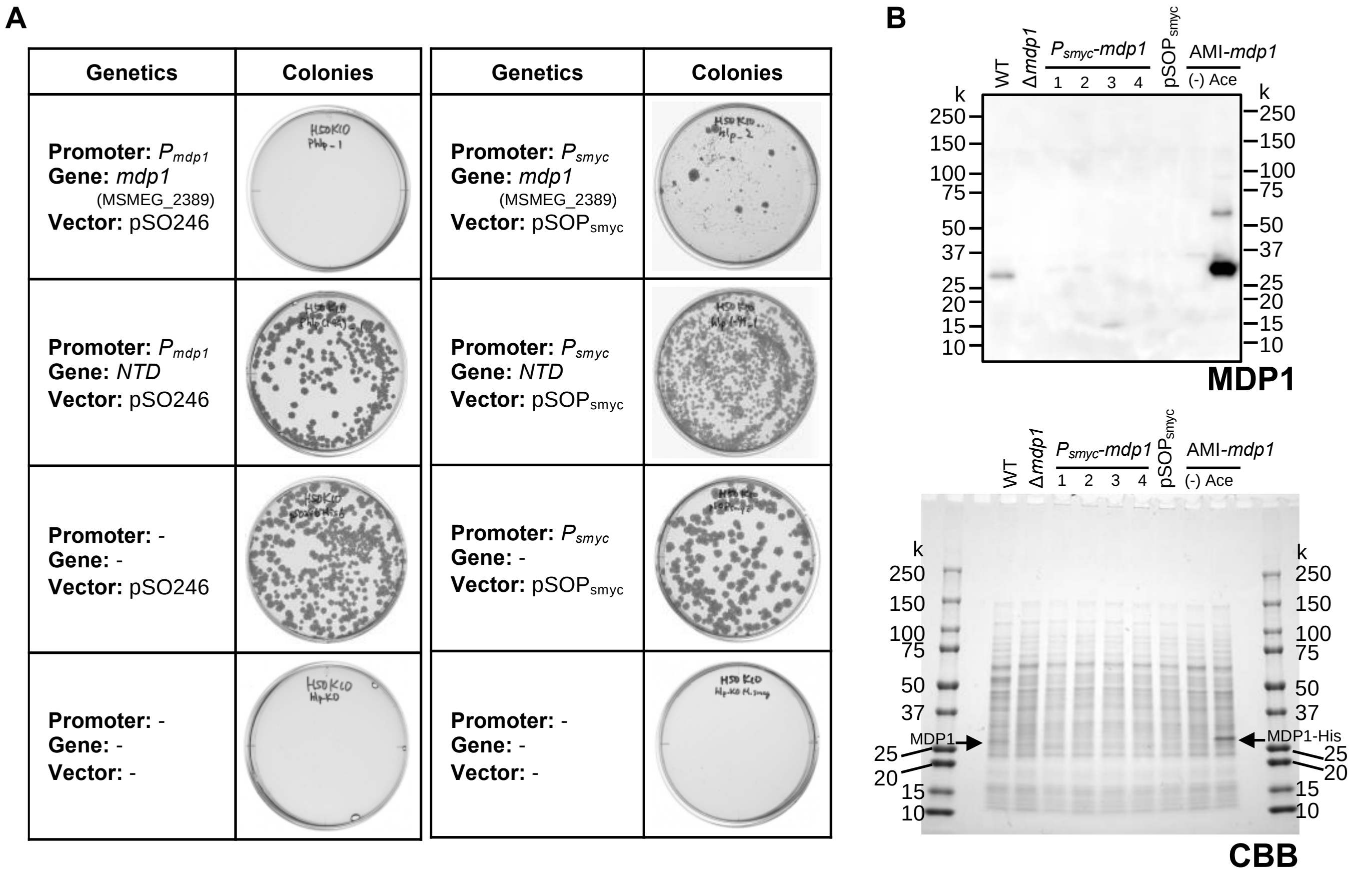

Supplementary Figure S2. Transformation of Δ*mdp1* with *mdp1* or *NTD* controlled by constitutively active promoters.

(**A**) Δ*mdp1* was transformed with pSO246 containing *M. smegmatis* MDP1 genes (*mdp1* and *NTD*) including a promoter region (P_{mdp1}) or with pSOP$_{smyc}$ containing *M. smegmatis* MDP1 genes downstream of P_{smyc} promoter[4]. Colonies which harbored the plasmid were selected on 7H11/OADC agar supplemented with 50 µg/ml Hyg and 10 µg/ml Km. Detailed procedures are described in the Supplementary information. (**B**) Expression levels of MDP1 in the individual isolates (P_{smyc}-*mdp1*, 1 - 4) were determined by SDS-PAGE stained with Coomassie Brilliant Blue R-250 (CBB) and western blotting using an anti-MDP1 antibody (MDP1)[5]. WT, Δ*mdp1*, and pSOP$_{smyc}$ represent wild-type and MDP1-defficient *M. smegmatis* strains, and Δ*mdp1* harboring pSOP$_{smyc}$, respectively. MDP1 expression of AMI-*mdp1* was induced in the presence (Ace) or absence (-) of 0.2% acetamide. The arrows indicate the positions of endogenous MDP1 of wild-type *M. smegmatis* and His-tagged MDP1 (MDP1 and MDP1-His, respectively).

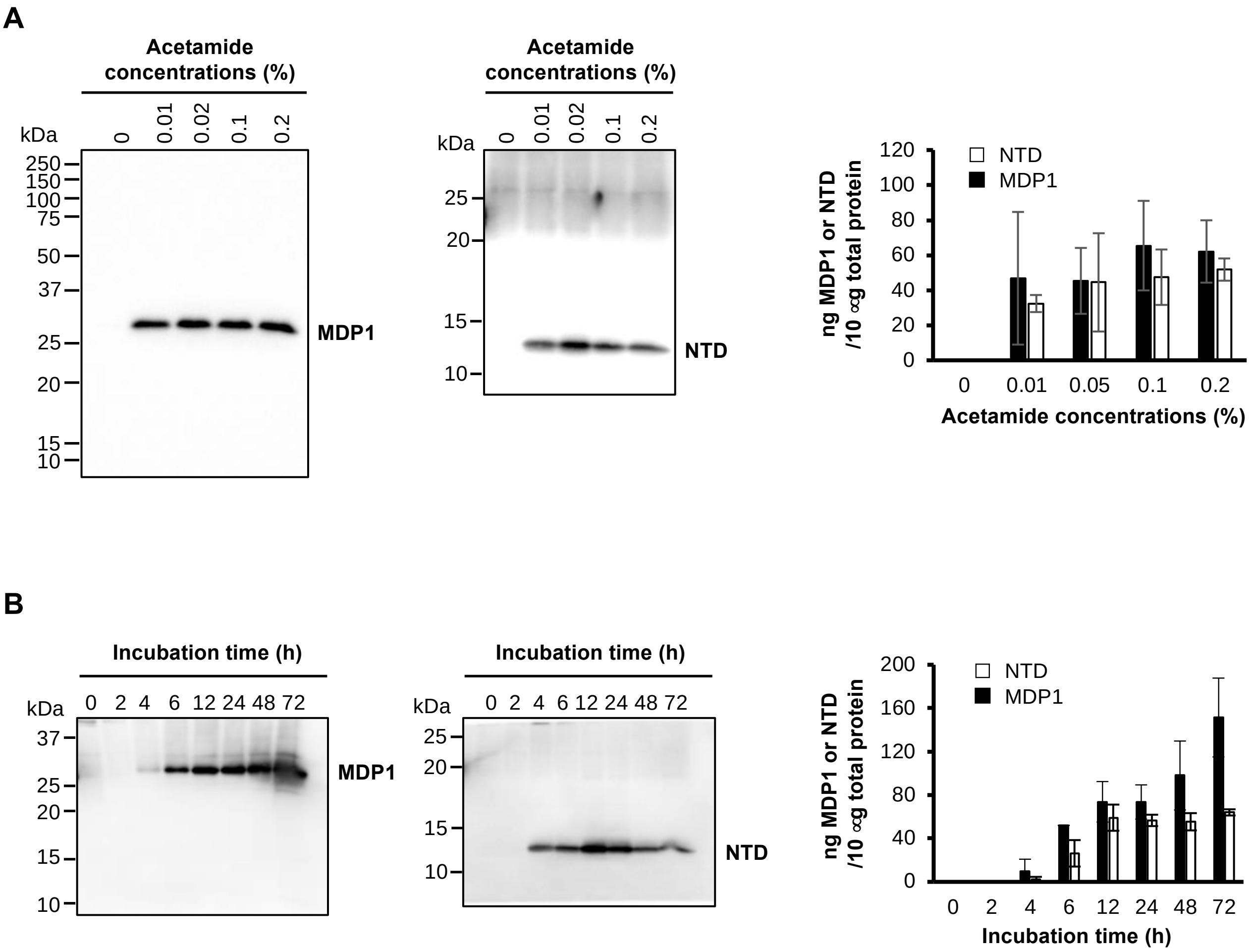

Supplementary Figure S3. Conditional induction of MDP1 and NTD expression.

(**A**) Intact MDP1 and NTD expression after incubation of AMI-*mdp1* and AMI-*NTD* respectively, with the indicated concentrations of Ace for 24 h as analyzed by western blotting. Full length western blots were presented. Positions of molecular weight markers were shown at the left side of each panel. Densitometric analysis of MDP1 (closed symbol) and NTD (open symbol) on western blots was also performed by CS Analyzer 3.0 software (ATTO, Tokyo, Japan). (**B**) Time-courses of MDP1 and NTD expression in the presence of 0.2% Ace as analyzed by western blotting. Full length western blots were presented. Densitometric analysis of MDP1 (closed symbol) and NTD (open symbol) proteins on western blots was further performed by CS Analyzer 3.0 software. Data shown is representative of at least three experiments.

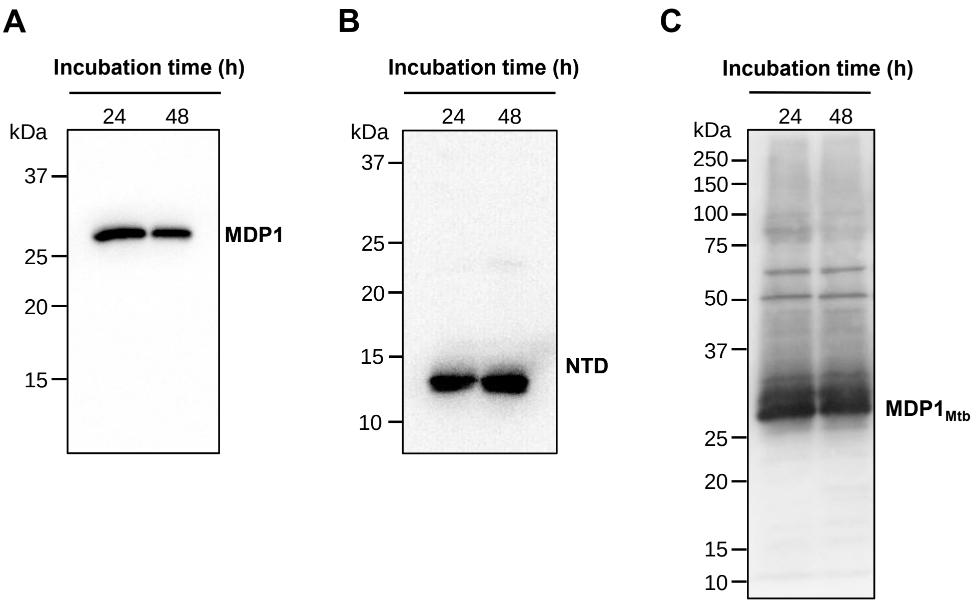

Supplementary Figure S4. The expression of MDP1, NTD, and MDP1$_{Mtb}$ in response to the addition of 0.2% Ace.

Full length western blots of Figure 2C and F were presented as Supplementary Figure S4. The expression of intact MDP1 (A), NTD (B), and MDP1$_{Mtb}$ (C) by AMI-*mdp1*, AMI-*NTD*, and AMI-*mdp1$_{Mtb}$*, respectively, 24 and 48 h after addition of 0.2% Ace were analyzed by western blotting. Positions of molecular weight markers were shown at the left side of each panel.

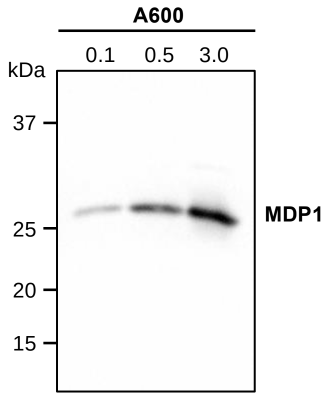

Supplementary Figure S5. The expression of endogenous MDP1 in wild-type *M. smegmatis* mc^2_155.

A full length western blot of Figure 7A was presented as Supplementary Figure S5. *M. smegmatis* mc^2_155 was harvested at the indicated A600 and MDP1 level was analyzed by western blotting. Positions of molecular weight markers were shown at the left side of a panel.

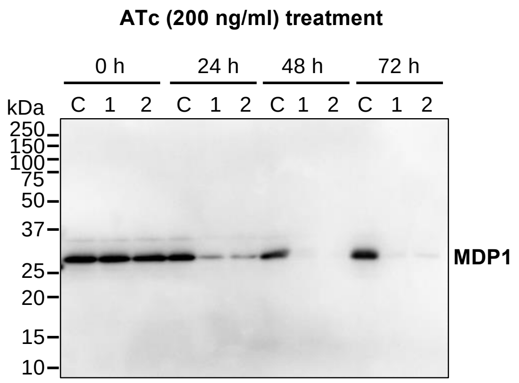

Supplementary Figure S6. Conditional reduction of MDP1 in *M. smegmatis.*

A full length western blot of Figure 8A was presented as Supplementary Figure S6. dCas9-mdp1_1 (1), dCas9-mdp1_2 (2), and dCas9 (C) were cultured in the presence of 200 ng/ml ATc and harvested at the indicated time. MDP1 levels were analyzed by western blotting. Positions of molecular weight markers were shown at the left side of a panel.

Supplementary information

Secondary structure prediction. Secondary structures of *M. smegmatis* MDP1 (MSMEG_2389) and *M. tuberculosis* $MDP1_{Mtb}$ (Rv2986c) were predicted using PSIPRED v3.3 of the PSIPRED Protein Sequence Analysis Workbench (http://bioinf.cs.ucl.ac.uk/psipred/)[2].

Cloning of MDP1 genes controlled by its own promoter or P_{smyc} promoter. First, a mycobacteria-*E. coli* shuttle vector pSO246[6] was modified to insert a His-tag sequence at the end of its multiple cloning site. Oligo-DNA fragments (5'-CGA TAC TAG TGG TAC CGG GCC CGC GGC CGC GCA CCA CCA CCA CCA CCA CTA AGT AC-3' and 5'-TTA GTG GTG GTG GTG GTG GTG CGC GGC CGC GGG CCC GGTA CCA CTA GTA T-3') which involve SpeI, KpnI, ApaI, SacII, NotI sites and a His-tag sequence were diluted in 40 mM Tris-HCl buffer (pH 8.0) containing 20 mM $MgCl_2$ and 50 mM NaCl. Reaction steps for annealing of these Oligo-DNA fragments were as follows: (i) heating at 95°C for 5 min and then (ii) cooling to 25°C for 90 min. Annealed DNA fragment was inserted between ClaI and KpnI sites. In this case, original KpnI site of pSO246 was deleted. Sequences encoding MDP1 (*mdp1*) and NTD (*NTD*) both including promoter region (P_{mdp1}) were amplified from *M. smegmatis* mc^2_155 genomic DNA by PCR using the primer sets: forward 5'-AGA GGA TCC CTG CGG AAA CTC GAT CAG AT-3' (common) and reverse 5'-TGC GCG GCC GCT TTG CGA CCC CGC CGA GCG GTT-3' for MDP1 or 5'-TGC GCG GCC GCT CCT TCT GCC GGG AGA CGC TGC-3' for NTD. Amplified DNA fragments (P_{mdp1}-*mdp1* and P_{mdp1}-*NTD*) were excised with BamHI and NotI and then inserted between BamHI and NotI sites of modified pSO246 (Km^R). Δ*mdp1* (Hyg^R) was then transformed with constructed plasmids and the resultant clones were selected on the agar plates containing 50 μg/ml Hyg and 10 μg/ml Km.

On the other hand, sequence encoding P_{smyc} promoter was amplified from pMC1[4] by PCR using the primer set: forward 5'-GAT AAC GTT GCT GAT TAG CTA AGC AGA AGG CCA-3' and reverse 5'-GGG GGA TCC CAT ATG GAA GTG ATT CCT CCT GAT GGT A-3'. Amplified DNA fragments was excised with Psp1406I and BamHI and then inserted between Psp1406I and BamHI sites of modified pSO246 (Km^R) above (designated as $pSOP_{smyc}$). Sequences encoding MDP1 (*mdp1*) and NTD (*NTD*) were amplified from *M.*

smegmatis mc^2_155 genomic DNA by PCR using the primer sets: forward 5’-TTT AAG CTT GTT CAT ATG AAC AAA GCG GAG CTC ATC GAC-3’ (common) and reverse 5’-TGC GCG GCC GCC CTG CGG CCC TTC TTG GCC GGG-3’ for MDP1 or 5’-TGC GCG GCC GCA CCA TCG GCC GGG AGC TTC TGT-3' for NTD. Amplified DNA fragments were excised with NdeI and NotI and then inserted between NdeI and NotI sites at the downstream of P_{smyc} promoter of $pSOP_{smyc}$ (Km^R). Δ*mdp1* (Hyg^R) was then transformed with constructed plasmids and the resultant clones were selected on the agar plates containing 50 µg/ml Hyg and 10 µg/ml Km.

References in Supplementary information

1. Enany, S. *et al.* Mycobacterial DNA-binding protein 1 is critical for long term survival of Mycobacterium smegmatis and simultaneously coordinates cellular functions. *Sci. Rep.* **7,** 6810 (2017).
2. Jones, D. T. Protein secondary structure prediction based on position-specific scoring matrices1. *J. Mol. Biol.* **292,** 195–202 (1999).
3. Bhowmick, T. *et al.* Targeting Mycobacterium tuberculosis nucleoid-associated protein HU with structure-based inhibitors. *Nat. Commun.* **5,** 4124 (2014).
4. Ehrt, S. *et al.* Controlling gene expression in mycobacteria with anhydrotetracycline and Tet repressor. *Nucleic Acids Res.* **33,** e21–e21 (2005).
5. Niki, M. *et al.* A novel mechanism of growth phase-dependent tolerance to isoniazid in mycobacteria. *J. Biol. Chem.* **287,** 27743–52 (2012).
6. Matsumoto, S. *et al.* A stable Escherichia coli-mycobacteria shuttle vector ‘pSO246’ in Mycobacterium bovis BCG. *FEMS Microbiol. Lett.* **135,** 237–243 (1996).
